# Personal

**Published:** 1894-08

**Authors:** 


### Personal.

DR. S. S. THORN, of Toledo, was elected president of the National Association of Railway Surgeons at its recent meeting in Galveston, Texas. Dr. Thorn was one of the founders of the association, is a surgeon of skill and judgment and is justly entitled to this honor so tardily bestowed.

---

DR. HORACE CLARK, of Buffalo, has resumed the special practice of laryngology at 21 North street. Hours, 9 to 1.

---

DR. V. P. GIBNEY has been appointed professor of clinical surgery in the College of Physicians and Surgeons, New York.

---

DR. G. FRANK LYDSTON, of Chicago, has been appointed medical director and surgeon in charge of the new Masonic Hospital that has been established on State street, in that city. Dr. Lydston is well known as a clever surgeon and a frequent contributor of valued articles to medical literature, and this appointment is well bestowed.

Dr. C. J. Reynolds, of Buffalo, has removed from 893 East Genesee street to the corner of Fox and High streets.

---

Dr. John B. Roberts has been elected president of the Pennsylvania State Medical Society. No better selection could have been made.

---

Dr. M. D. Mann, of Buffalo, was elected president of the American Gynecological Society at its late meeting in Washington. This is the first time this office has been given outside of the inner circle of the first generation of gynecologists.

---

Dr. and Mrs. A. A. Hubbell and daughter, of Buffalo, sailed for Europe, on the steamer Lucania, on Saturday, July 28, 1894. Dr. Hubbell will attend the International Ophthalmological Congress in Edinburgh in August, and, with Mrs. Hubbell and daughter, will spend altogether about six weeks abroad for much needed rest and recreation.

---

Dr. Jane W. Carroll, of Buffalo, has removed her office from Cherry street to 489 East Genesee street. Dr. Carroll's daughter, Dr. Evangeline Carroll, has returned from Detroit, where she was resident physician at the Woman's Hospital, and has established her office also at 489 East Genesee street.

---

Dr. John Williams, of 63 Brook street, London, who attended the Duchess of York during her recent confinement, has been made a baronet by the Queen. Sir John Williams is an honorary fellow of the American Association of Obstetricians and Gynecologists.

---

Dr. J. B. S. Holmes, formerly of Rome, Ga., has removed to Atlanta, where he is building a private hospital for women that will be ready for patients on September 1, 1894. Dr. Holmes has also been appointed professor of obstetrics and gynecology in the Southern Medical College.

---

Dr. I. S. Stone, of Washington, D. C., publishes in the *Virginia Medical Monthly*, May, 1894, a series of abdominal sections—the second series of twenty-five in his second hundred sections. But one death occurred in the present series, in which case a long history of severe suppurative disease presented. Pus was found in the pelvis of one kidney at the autopsy. Not an ounce of urine was secreted during the twenty-four hours the patient lived after the operation. Dr. Stone's work is highly creditable to himself and to his profession.

